# Decrotonylation of cGAS K254 prompts homologous recombination repair by blocking its DNA binding and releasing PARP1

**DOI:** 10.1016/j.jbc.2024.107554

**Published:** 2024-07-11

**Authors:** Hejiang Guo, Yang Han, Shibo Yao, Bijia Chen, Hongling Zhao, Jin Jia, Shi Chen, Yuhao Liu, Shanshan Gao, Hua Guan, Jun Lu, Ping-Kun Zhou

**Affiliations:** 1 Department of Radiation Biology, Beijing Key Laboratory for Radiation Biology, Beijing Institute of Radiation Medicine, Beijing, China; 2 School of Public Health, Hengyang Medical School, University of South China, Hengyang, Hunan Province, China; 3 Department of Medical Oncology, Beijing YouAn Hospital, Laboratory for Clinical Medicine, Capital Medical University, Beijing, China

**Keywords:** cGAS, crotonylation, HR repair, SIRT3, PARP1

## Abstract

Cyclic GMP-AMP synthase (cGAS), a cytosolic DNA sensor, also exhibits nuclear genomic localization and is involved in DNA damage signaling. In this study, we investigated the impact of cGAS crotonylation on the regulation of the DNA damage response, particularly homologous recombination repair, following exposure to ionizing radiation (IR). Lysine 254 of cGAS is constitutively crotonylated by the CREB-binding protein; however, IR-induced DNA damage triggers sirtuin 3 (SIRT3)-mediated decrotonylation. Lysine 254 decrotonylation decreased the DNA-binding affinity of cGAS and inhibited its interaction with PARP1, promoting homologous recombination repair. Moreover, SIRT3 suppression led to homologous recombination repair inhibition and markedly sensitized cancer cells to IR and DNA-damaging chemicals, highlighting SIRT3 as a potential target for cancer therapy. Overall, this study revealed the crucial role of cGAS crotonylation in the DNA damage response. Furthermore, we propose that modulating cGAS and SIRT3 activities could be potential strategies for cancer therapy.

The cellular genome is continuously exposed to endogenous and exogenous genotoxic stresses that can damage DNA. Unrepaired DNA damage can trigger a series of deleterious biological events, such as apoptosis, genomic instability, and carcinogenesis (1). Ionizing radiation (IR) is a major cause of double-strand DNA breaks (DSBs), which are highly deleterious lesions (2, 3). In response to DSBs, cells activate specific DNA repair pathways, including nonhomologous end joining (NHEJ) and homologous recombination (HR), to maintain genetic stability (4, 5, 6). NHEJ, an error-prone DNA repair pathway initiated by the binding of the DNA–PK complex to DSB sites, remains active throughout the cell cycle (7, 8) and involves the ligation of DNA break ends, often resulting in indel mutations (9). In contrast, the HR repair pathway uses a template-guided DNA extension mechanism to accurately restore the sequence at damaged sites (10).

cGAS (cyclic GMP-AMP synthase), a well-known cytosolic DNA sensor involved in innate immune reactions (11, 12), is a nucleotidyltransferase comprising an unstructured N-terminal domain and a structured C-terminal catalytic domain (13, 14). The latter contains a zinc-binding dimerization motif and two DNA-binding sites, each covering 16 to 18 base pairs (15, 16, 17). Previous studies have revealed the intersection between the DNA damage response (DDR) and immune response (18, 19, 20, 21), with cGAS playing multifaceted roles in cellular processes, particularly in DNA damage repair. It acts not only as a sentinel in innate immunity but also significantly influences DNA repair and cellular responses to genotoxic stress (22, 23, 24, 25, 26). Evidence shows that cGAS directly participates in DNA damage repair through its interaction with PARP1, which disrupts the association of PARP1 with the DSB repair factor Timeless, thereby impairing the HR repair pathway (27). Additionally, cGAS dimerization promotes chromatin compaction, affecting RAD51-mediated strand invasion and impairing D-loop formation during HR repair (28). Beyond these mechanisms, cGAS is crucial in maintaining genome stability by interacting with the replication fork and modulating replication dynamics (29). cGAS function is regulated through various posttranslational modifications (PTMs) (30, 31, 32, 33, 34, 35). For instance, RIOX1 removes monomethylation at K491 of cGAS, facilitating its interaction with PARP1 and blocking Timeless recruitment (36). PTMs are likely the primary regulatory mechanisms affecting cGAS activity and function in the DDR.

Lysine crotonylation (Kcr) is a recently identified protein modification in which a crotonyl group (-CH2-CH =CH-CO-) is added to lysine residues of a protein (37). This modification, like others, is tightly regulated by specific enzymes, namely crotonylases and decrotonylases (38, 39, 40, 41, 42, 43). Although Kcr is evolutionarily conserved and abundantly present across species (44, 45, 46, 47, 48), its functional roles are still being elucidated. One study revealed a temporary reduction in H3K9cr levels following laser microirradiation–induced DNA damage, suggesting for the first time a potential role for Kcr in the DDR (49). CDYL1 crotonyl-CoA hydratase activity causes the removal of the transcriptional elongation factor, possibly impacting gene transcription in the DNA lesion (50, 51). Kcr is also expressed in nonhistone proteins, including those involved in the DDR. RPA1 Kcr is crucial for its interactions with single-strand DNA and key HR-mediated DSB repair-promoting factors (52).

Despite recent advances in understanding the effects of IR on human health and its potential to eradicate tumor cells, research on the role of protein modifications like crotonylation in IR-induced DNA damage repair remains limited. In this study, we investigated the dynamic role of cGAS crotonylation and its impact on the DDR. We found that cGAS is naturally crotonylated, with its crotonylation levels significantly reduced by irradiation. Specifically, lysine 254 was identified as a crucial crotonylation site that mediates cGAS's involvement in the DDR. Sirtuin 3 (SIRT3) regulates the decrotonylation of cGAS at this site, inhibiting cGAS's ability to bind DNA. This alteration impacts cGAS's interaction with PARP1 and reduces its recruitment to DSB sites, thereby relieving the suppression of HR repair. Furthermore, knocking down SIRT3 results in activation defects in the HR pathway, impairing DNA repair and increasing the sensitivity of tumor cells to DNA-damaging agents. These findings provide valuable insights into potential new therapeutic targets for diseases associated with immune dysregulation and genomic instability.

## Results

### Identification of cGAS crotonylation in response to radiation-induced DNA damage

**Figure 1:**
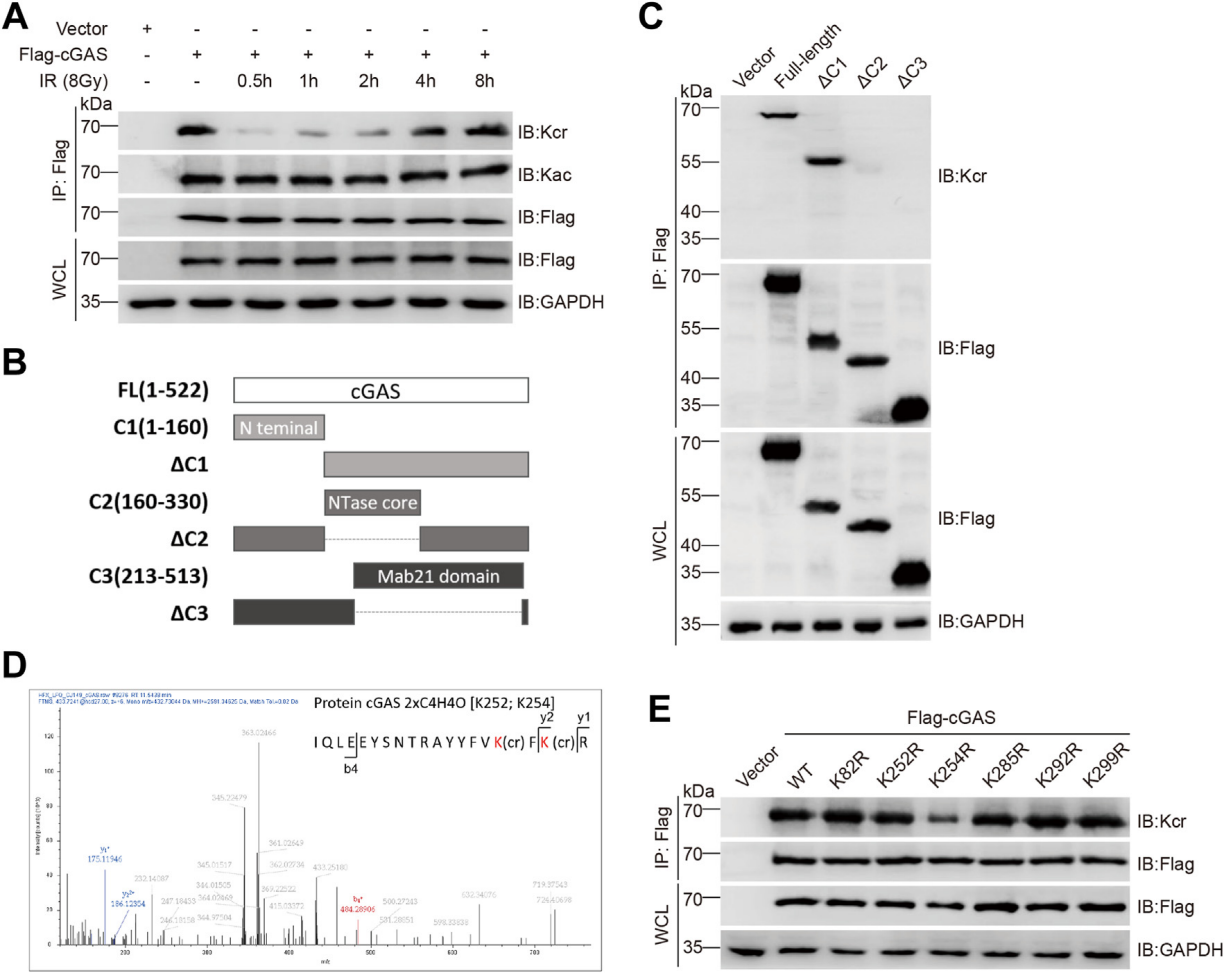
**Identification of cGAS crotonylation.***A*, crotonylation of the cGAS protein and the effect of γ-ray irradiation. Immunoblotting (IB) analyses were performed on whole-cell lysates (WCLs) and anti-Flag immunoprecipitates (IPs) from HeLa cells transfected with the Flag-cGAS or Flag-vector plasmid. cGAS crotonylation and acetylation were determined at the indicated times post-IR (8 Gy γ-ray). *B*, schematic diagrams of cGAS and its truncated mutants. *C*, IB analysis was performed on WCLs and anti-Flag IPs of HeLa cells transfected with plasmids encoding either full-length Flag-cGAS or its truncation mutants, and the crotonylation levels were determined. *D*, mass spectrometry analysis of cGAS crotonylation sites. The spectrum displays the mass-to-charge (m/z) ratios corresponding to peptide fragments containing crotonylated lysine residues, pinpointing the precise modification sites. *E*, IB analysis was performed on WCLs and anti-Flag IPs of HeLa cells transfected with the indicated Flag-cGAS mutants to detect crotonylation levels. cGAS, cyclic GMP-AMP synthase.

Although cGAS is recognized as a cytosolic DNA sensor, it was recently found to localize in the nucleus to block IR-induced HR repair of DNA DSBs (27, 28, 36), and various forms of PTMs were reported to affect cGAS activity. Crotonylation has been shown to play an important role in the regulation of DSBs repair, and we speculated that cGAS might also be modified by crotonylation. Immunoprecipitation (IP) assays revealed constitutive crotonylation of cGAS in nonirradiated cells, which was markedly decreased form ∼0.5 to 2 h postirradiation, a critical period of initiating DNA repair. Whereas, the acetylation levels of cGAS did not significantly change after irradiation (Fig. 1*A*).

The highly basic unstructured N-terminal (1–160 aa) of cGAS is essential for promoting liquid-phase condensation and is critical for cGAS activation; the nucleotidyl transferase (NTase) domain (161–330 aa) is pivotal for the enzymatic function of cGAS; and the male abnormal 21 (Mab21) domain (213–522 aa) can insert into the minor groove of DNA and interact with the sugar-phosphate backbone. To identify which cGAS domain is critical for crotonylation, we constructed three truncated forms of cGAS (Fig. 1*B*), with deletions of the N-terminal, NTase, and Mab21 domains for IP assays, and found that crotonylation did not occur in either truncated cGAS mutant with the deletion of the NTase or Mab21 (▵C2 and ▵C3), indicating that cGAS crotonylation mainly occurs in these two domains (Fig. 1*C*).

High-resolution liquid chromatography-tandem mass spectrometry analysis of cGAS IP samples for site specificity identified six Kcr sites: K82, K252, K254, K285, K292, and K299 (Figs. 1*D* and S1, *A* and *B*). IP of cGAS mutants with lysine-to-arginine substitutions at one of these sites revealed reduced crotonylation in the K254R mutant compared to that in wildtype (WT) cGAS (Fig. 1*E*), suggesting that K254 is the dominant crotonylation site.

### CREB-binding protein mediates cGAS crotonylation

**Figure 2:**
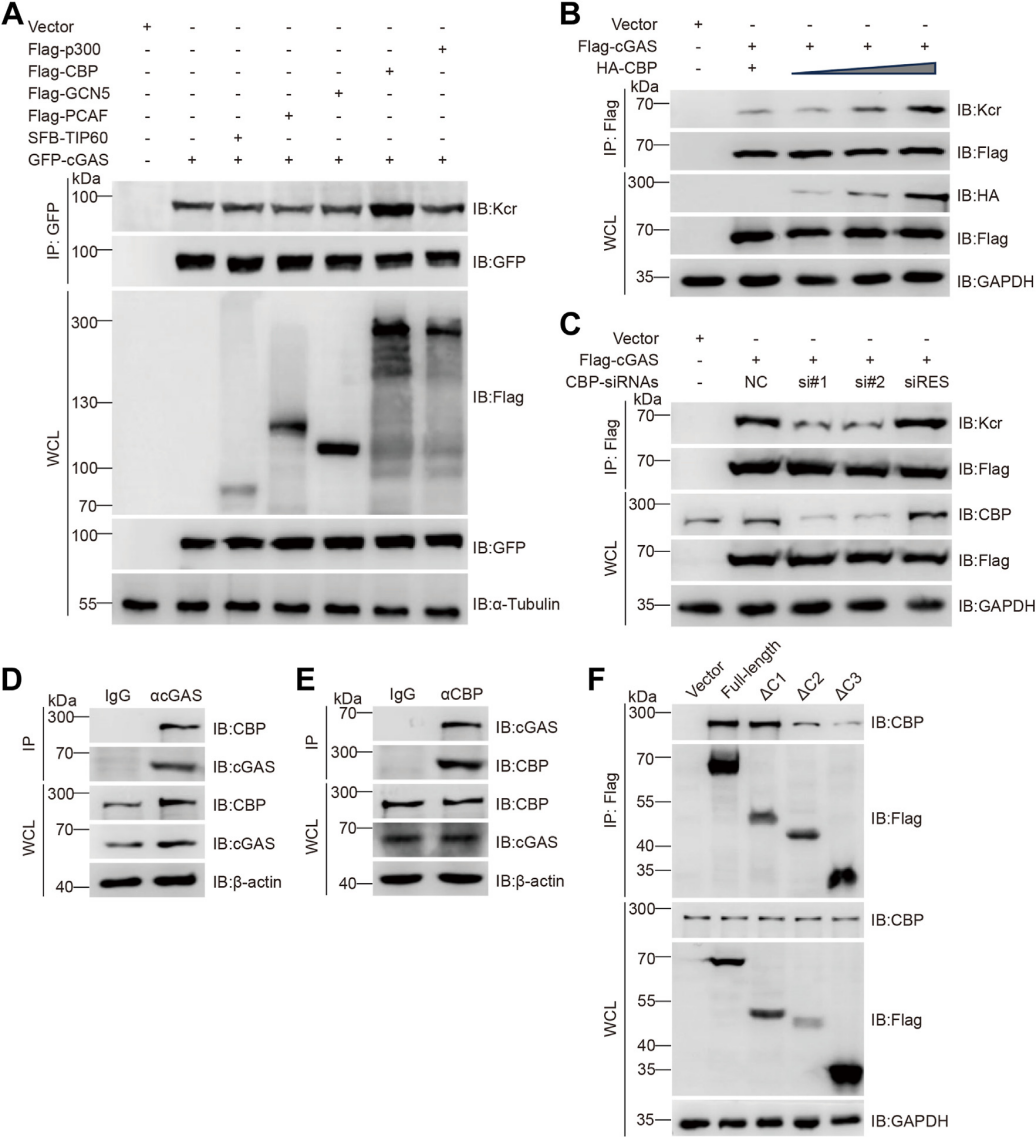
**CBP mediates cGAS crotonylation.***A*, IB analysis was performed on WCLs and anti-GFP IPs of HEK293T cells transfected with the indicated constructs. *B*, IB analysis was performed on WCLs and anti-Flag IPs of HeLa cells transfected with increasing amounts of HA-CBP plasmid (1 μg, 2 μg, or 4 μg). *C*, IB analysis was performed on WCLs and anti-Flag IPs of HeLa cells transfected with the indicated siRNA and plasmids. *D* and *E*, Co-IP of endogenous cGAS with CBP from HeLa cells. CBP and cGAS were immunoprecipitated using anti-cGAS and anti-CBP antibodies, respectively. *F*, IB analysis was performed on WCLs and anti-Flag IPs of HeLa cells transfected with plasmids encoding full-length Flag-cGAS and Flag-cGAS truncation mutants, and interactions with CBP were detected. CBP, CREB-binding protein; cGAS, cyclic GMP-AMP synthase; IB, immunoblotting; IP, immunoprecipitate; WCLs, whole-cell lysates.

Enzymes that regulate lysine acetylation also appear to regulate Kcr (39, 40, 41, 42, 43). Analysis of HEK293T cells transfected with a series of plasmids encoding different acetyltransferases showed that CREB-binding protein (CBP) was associated with the highest crotonylation level among all groups transfected with the plasmids harboring the indicated acetyltransferase genes (Fig. 2*A*). cGAS Kcr progressively increased with increasing CBP transfection (Fig. 2*B*). Moreover, knocking down CBP in HeLa cells using small-interfering RNAs (siRNAs) significantly decreased cGAS crotonylation, and CBP supplementation restored it (Fig. 2*C*).

Co-immunoprecipitation (Co-IP) and Western blotting analyses of HeLa cells with anti-cGAS or anti-CBP antibodies demonstrated endogenous interactions between CBP and cGAS (Fig. 2, *D* and *E*). Further analyses with truncated cGAS mutants revealed that the interactions between CBP and the NTase (ΔC2)- or Mab21 (ΔC3)-truncated cGAS mutants were significantly reduced compared to the WT protein (Fig. 2*F*). This suggests that the interactions primarily occur within the NTase or Mab21 regions of cGAS, which are consistent with the domains involved in crotonylation.

### SIRT3 acts as a decrotonylase to erase the crotonylation of cGAS

**Figure 3:**
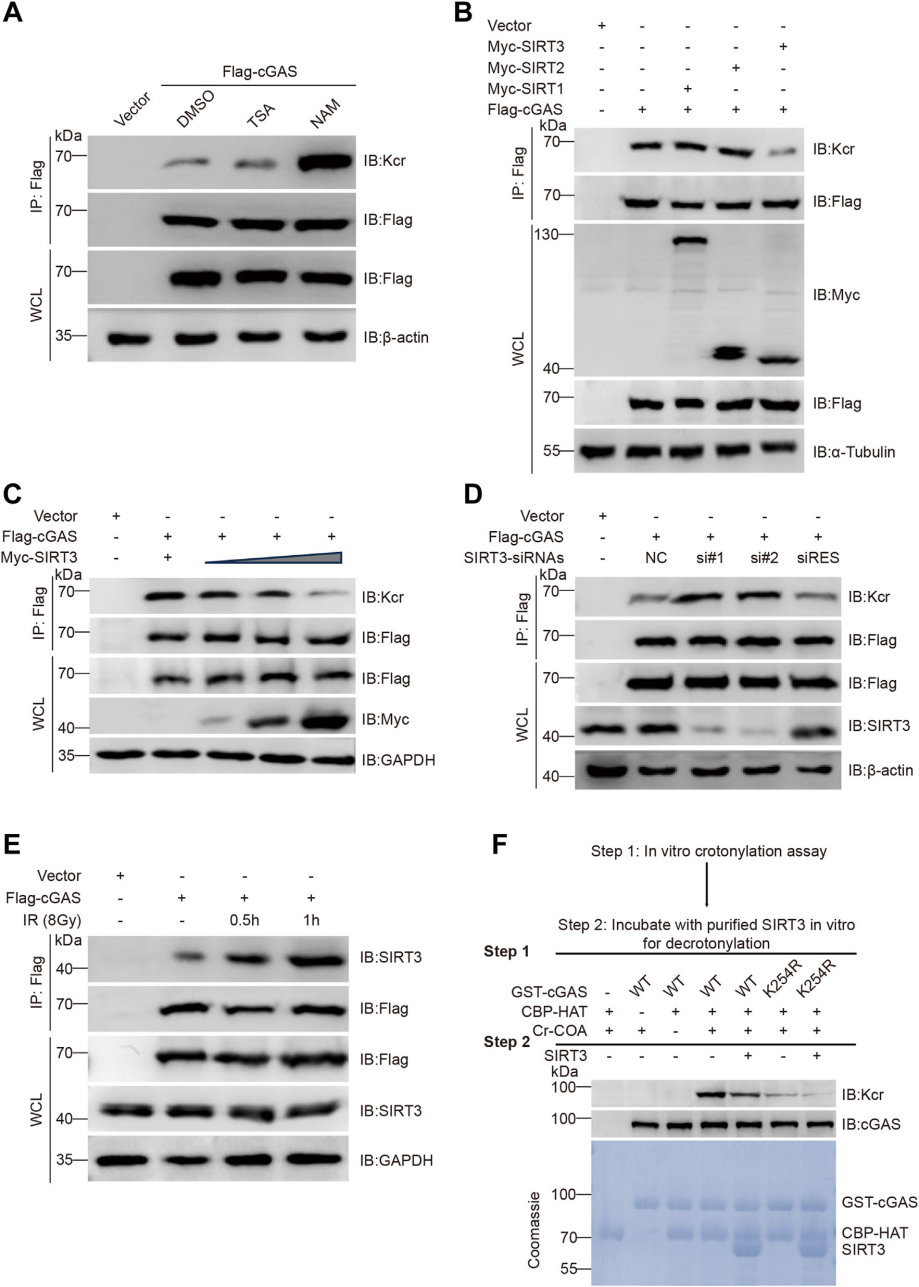
**SIRT3 mediates cGAS decrotonylation.***A*, IB analysis was performed on WCLs and anti-Flag IPs of HeLa cells transfected with the Flag-cGAS plasmid and pretreated with 1 μM TSA or 10 mM NAM for 10 h to observe the effects on cGAS crotonylation levels. *B*, IB analysis was performed on WCLs and anti-GFP IPs of HeLa cells transfected with the indicated constructs. *C*, IB analysis was performed on WCLs and anti-Flag IPs of HeLa cells transfected with increasing amounts of Myc-SIRT3 plasmid (1 μg, 2 μg, or 4 μg). *D*, IB analysis was performed on WCLs and anti-Flag IPs of HeLa cells transfected with the indicated siRNAs and plasmids. *E*, Co-IP of endogenous SIRT3 with Flag-cGAS from HeLa cells at the indicated times post-IR (8 Gy γ-ray). *F*, *in vitro* cell-free reaction assay of cGAS crotonylation and decrotonylation by CBP and SIRT3, respectively. Workflow of the *in vitro* assay (*upper panel*). The samples were analyzed by IB. cGAS, cyclic GMP-AMP synthase; IB, immunoblotting; IP, immunoprecipitate; NAM, nicotinamide; TSA, trichostatin A; WCLs, whole-cell lysates.

It has been reported that some deacetylases also have decrotonylase activity (38, 39, 40, 41, 42, 43). To determine the decrotonylation of cGAS, we detected the crotonylation of exogenously expressed cGAS in HeLa cells treated with trichostatin A (TSA), an inhibitor of the HDAC family deacetylases, and nicotinamide (NAM), an inhibitor of the SIRT family deacetylases. Treatment with NAM resulted in a significant increase in cGAS crotonylation, while TSA treatment had little effect (Fig. 3*A*), indicating that the decrotonylase of cGAS belongs to the SIRT family.

Further experiments with various SIRT family deacetylases expressed in HeLa cells demonstrated that overexpression of SIRT3 significantly reduced cGAS crotonylation (Fig. 3*B*). cGAS Kcr progressively decreased with increasing SIRT3 expression (Fig. 3*C*). Moreover, knockdown of SIRT3 using siRNAs led to increased cGAS crotonylation, which was normalized upon re-expression of SIRT3 (Fig. 3*D*).

Following irradiation, the interaction between cGAS and SIRT3 increased (Fig. 3*E*). In contrast, overexpression of the cGAS Y215E mutant did not show any interaction with SIRT3 (Fig. S2*A*), suggesting that the nuclear translocation of cGAS in response to DNA damage is essential for its interaction with SIRT3. We purified the cGAS protein and the cGAS K254R mutant protein from yeast. An *in vitro* crotonylation assay with crotonyl-CoA, using CBP or SIRT3 as cofactors, showed that purified WT cGAS could be crotonylated. In this assay, CBP increased crotonylation while SIRT3 decreased it. However, the cGAS K254R mutant showed significantly reduced CBP-mediated crotonylation (Fig. 3*F*). Taken together, these results collectively suggest that SIRT3 acts as the primary decrotonylase for cGAS and indicate that CBP and SIRT3 coregulate cGAS K254 crotonylation.

### SIRT3-mediated decrotonylation inhibits cGAS binding to DNA

**Figure 4:**
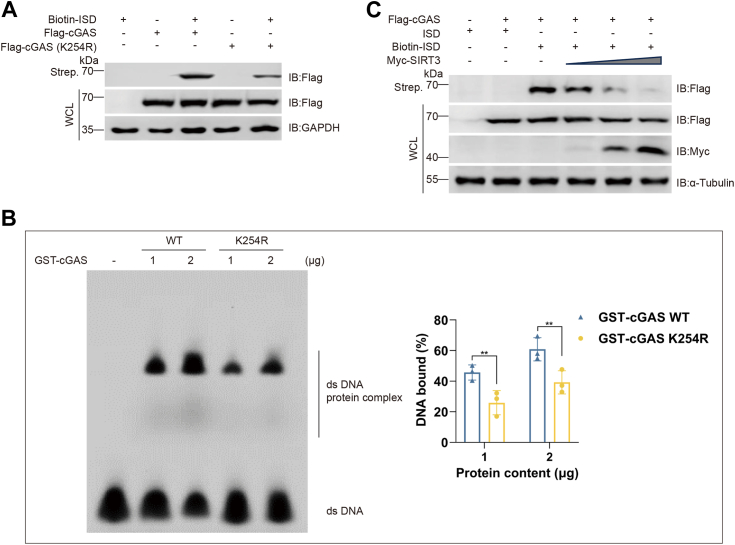
**Decrotonylation inhibits cGAS binding to DNA.***A*, HEK293T cells were transfected with the indicated plasmids. DNA pull-down assay to measure the DNA binding ability of WT and K254R cGAS. *B*, EMSA assay was performed with double-stranded DNA and the cGAS WT and K254R mutant proteins. The data are presented as the means ± SDs of triplicate experiments. ∗∗*p* < 0.01. *C*, HEK293T cells were transfected with the indicated plasmids. Biotin-ISD was transfected into cells 6 h before harvesting. Lysates were coprecipitated with streptavidin beads and later analyzed by IB. cGAS, cyclic GMP-AMP synthase; EMSA, electrophoretic mobility shift assay; IB, immunoblotting.

We further investigated the functional relevance of cGAS crotonylation in nuclear genomic DNA sensing. K254 is located in the DNA-binding B site of cGAS, and this region is critical for its interaction with double-strand DNA, according to a previous report (16). We synthesized 45-bp fragments of IFN-stimulatory DNA (ISD), a commonly utilized stimulator for cGAS, and conducted a DNA-binding assay. Results indicated that cGAS K254R exhibited significantly reduced binding affinity to biotin-labeled ISD compared to cGAS WT (Fig.4*A*), and an electrophoretic mobility shift assay (EMSA) further substantiated this finding (Fig. 4*B*).

As SIRT3 was identified as the decrotonylase of cGAS in this study, we tested whether SIRT3 influences the DNA-binding activity of cGAS. HEK293T cells were transfected with biotin-labeled ISDs, and a DNA pull-down assay was performed to precipitate DNA-bound Flag-cGAS. Following the overexpression of Flag-cGAS, streptavidin agarose beads were used to capture the biotin-labeled ISD. As depicted in Figure 4*C*, cGAS demonstrated binding to the biotin-labeled ISD. However, this binding capacity progressively decreased with increasing expression of SIRT3, suggesting that SIRT3 disrupts the formation of the cGAS–DNA–binding complex (Fig. 4*C*). Collectively, these findings suggest that cGAS crotonylation is essential for its ability to bind to double-stranded DNA.

### Decrotonylation inhibits cGAS interaction with PARP1 and promotes HR

**Figure 5:**
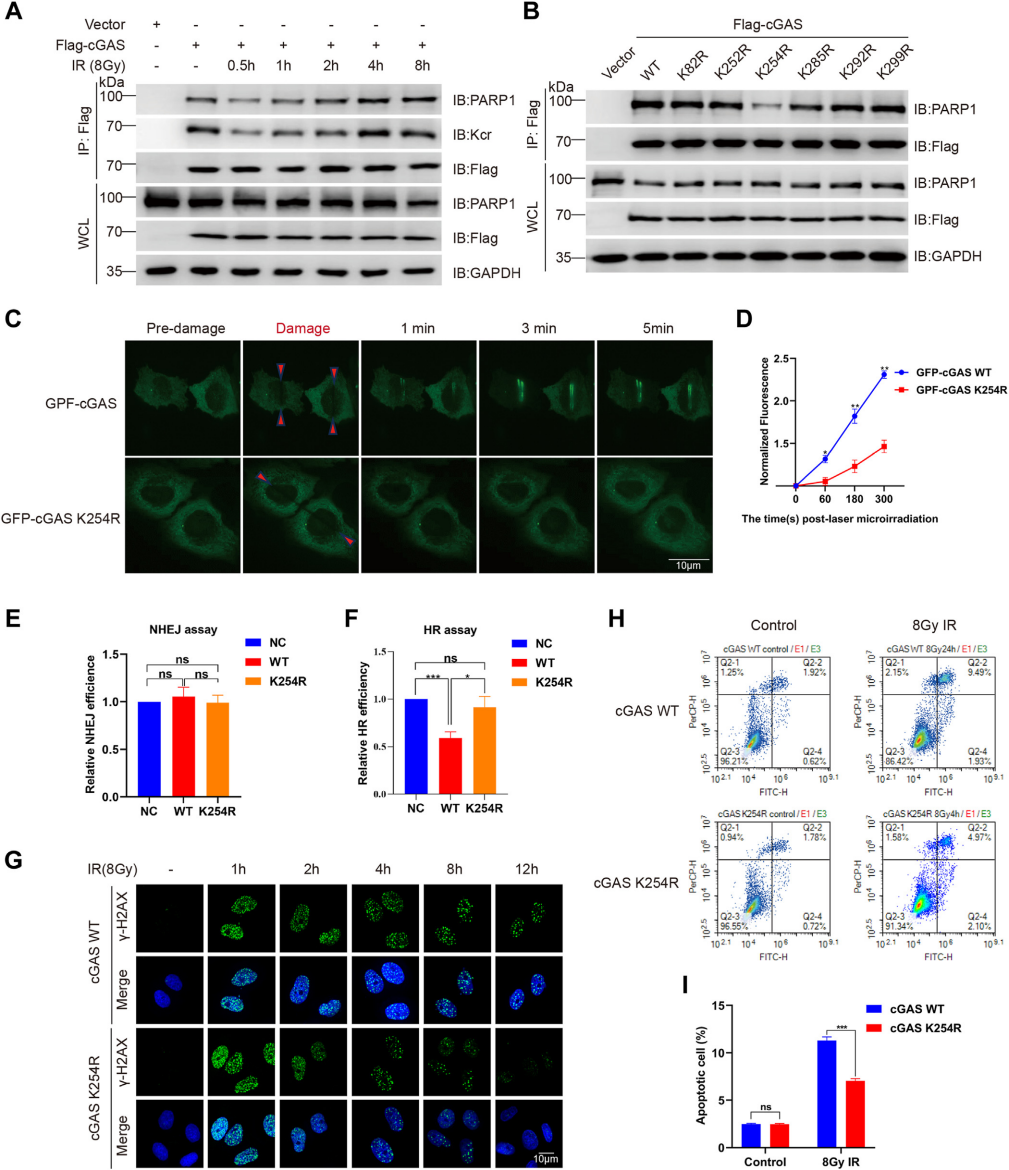
**Decrotonylation of K254 relieves PARP1–cGAS interaction and promotes homologous recombination.***A*, IB analysis was performed on WCLs and anti-Flag IPs of HeLa cells. Cells were analyzed for cGAS crotonylation and interaction with PARP1 at the indicated times post-IR (8 Gy γ-ray). *B*, IB analysis was performed on WCLs and anti-Flag IPs of HeLa cells transfected with the indicated Flag-cGAS mutants to detect interactions with PARP1. *C*, HeLa cells transfected with plasmids encoding GFP-cGAS WT or K254R were subjected to laser microirradiation. *D*, GFP bands were quantified. The average value obtained from 10 replicates was measured and analyzed. Error bars represent the standard deviation: ∗*p* < 0.05, ∗∗*p* < 0.01. *E*, EJ5-GFP reporter assay was used to examine NHEJ efficiency, as explained in the “Experimental procedures” section. The representative data are shown as the means ±SDs of triplicate experiments. *F*, DR-GFP reporter assay was performed to examine the HR efficiency, as described in the “Experimental procedures” section. The represented data are shown as the means ±SDs of triplicate experiments. ∗*p* < 0.05, ∗∗∗*p* < 0.001. *G*, representative images of γ-H2AX foci in cGAS WT and cGAS K254R mutant cell lines at different time points (1 h, 2 h, 4 h, 8 h, and 12 h) postirradiation. Cells were fixed and stained with an anti-γ-H2AX antibody to visualize DNA damage foci. *H*, flow cytometric histograms of apoptosis detection. cGAS WT and K254R mutant HeLa cells were treated with 8 Gy γ-ray irradiation. Apoptosis was detected at 24 h postirradiation. *I*, quantification of IR-induced apoptosis in cGAS WT and cGAS K254R mutant cells. The data are shown as the means ± SDs of triplicate experiments. ∗∗∗*p* < 0.001, as compared with the cGAS WT group. cGAS, cyclic GMP-AMP synthase; HR, homologous recombination; IB, immunoblotting; IPs, immunoprecipitates; IR, ionizing radiation; NHEJ, nonhomologous end joining; ns, nonsignificant; WCLs, whole-cell lysates.

A previous report (27) demonstrated interactions between cGAS and PARP1 and that impaired PARP1-mediated HR repair, and the association of cGAS with PARP1 occurs *via* nucleic acid binding. We asked whether the interaction of cGAS with PARP1 is associated with cGAS crotonylation in response to DNA damage. Co-IP assays showed that the interaction between cGAS and PARP1 decreased significantly within 0.5 to 2 h postirradiation and later recovered, mirroring the changes in cGAS crotonylation levels after irradiation (Fig. 5*A*). Moreover, compared with the WT cGAS, the cGAS K254R mutant exhibited reduced interaction with PARP1 (Fig. 5*B*).

In HeLa cells, a laser microirradiation-induced DNA DSBs assay showed that the K254R mutation impaired the recruitment of GFP-cGAS to DNA damage sites (Fig. 5, *C* and *D*).

Next, we generated the cGAS WT and cGAS K254R mutated cell lines. Our findings indicate that while neither the cGAS WT nor the cGAS K254R mutant significantly affected the NHEJ pathway (Figs. 5*E* and S3*A*), the WT cGAS significantly inhibited HR repair. In contrast, the cGAS K254R did not impact HR repair (Figs. 5*F* and S3*B*), suggesting that the K254R mutation alleviates the suppression of HR repair typically mediated by WT cGAS. Additionally, the reduction of γ-H2AX foci, a marker of DNA damage, occurred more rapidly in the cGAS K254R mutant cell lines (Figs. 5*G* and S3*C*), indicating a faster resolution of DNA DSBs and an alleviation of DNA-repair deficiencies. The IR-induced apoptosis of cGAS K254R mutant cells decreased markedly compared to that of cGAS WT cells (Fig. 5, *H* and *I*). These results indicate that K254 decrotonylation relieves the interaction between PARP1 and cGAS to promote its participation in HR.

### SIRT3 deficiency impairs DNA repair and prevents HR repair

**Figure 6:**
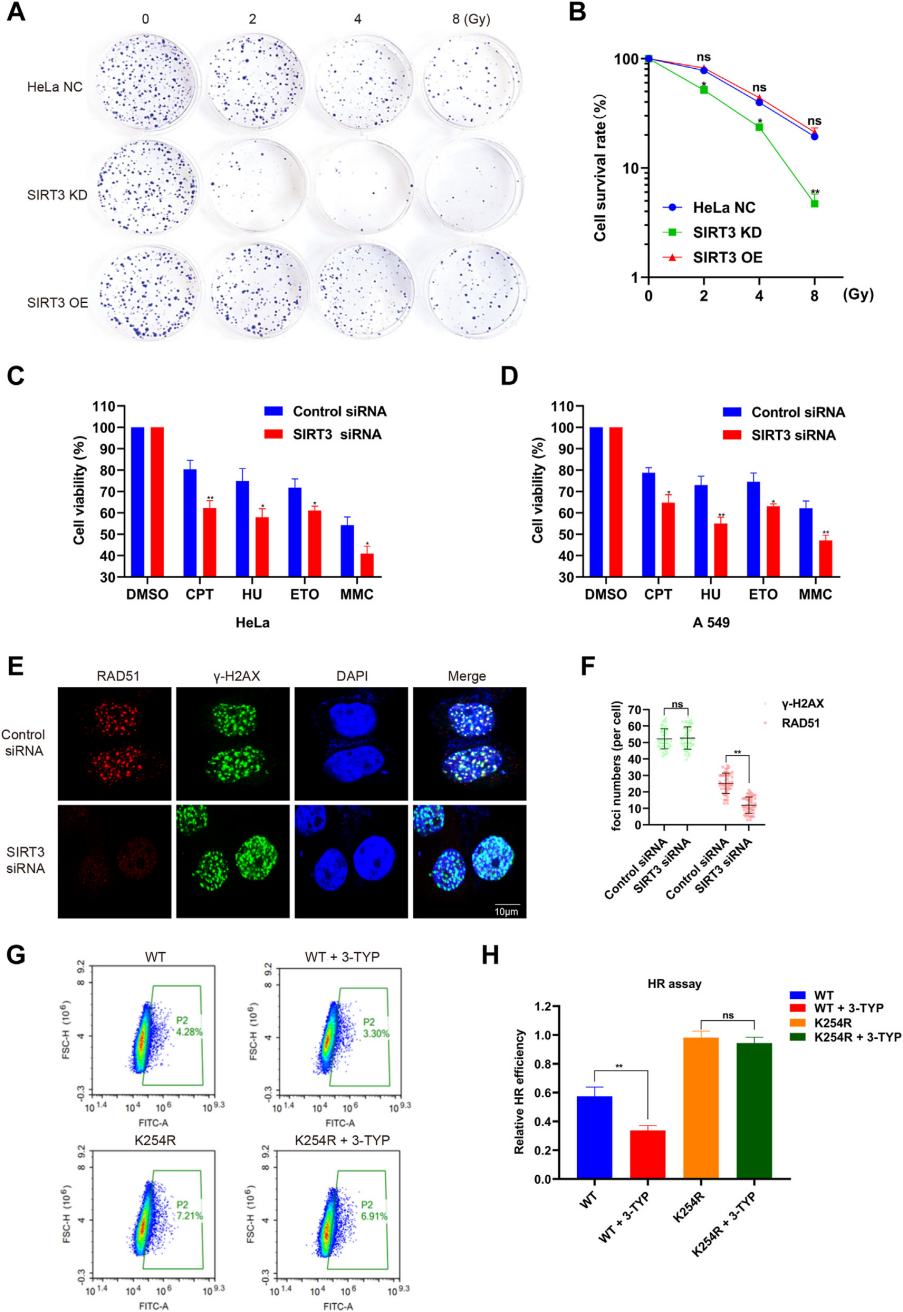
**SIRT3 deficiency impairs DNA repair and prevents HR repair.***A*, survival of HeLa cells in different groups after 0, 2, 4, and 8 Gy irradiation was detected by colony formation assay. *B*, HeLa cells were transfected with the indicated siRNAs, and later formation assays were conducted. The data are presented as the means ± SDs of triplicate experiments. ∗*p* < 0.05, ∗∗*p* < 0.01. *C* and *D*, sensitivities of SIRT3-depleted HeLa cells (*C*) and MCF-7 cells (*D*) to DNA damage or replication stress–inducing agents were determined by MTS assays. The data are shown as the means ± SDs of biological triplicates. ∗*p* < 0.05, ∗∗*p* < 0.01. *E* and *F*, HeLa cells were transfected with the indicated SIRT3 siRNAs. After 24 h, cells were treated with IR (8 Gy) for 1 h. An immunofluorescence assay was performed to test the extent of RAD51 foci formation using the corresponding antibodies. Scale bar, 10 μm. The data are presented as the means ± SDs of triplicate experiments. ∗∗*p* < 0.01. *G*, flow cytometry analysis of HR repair efficiency in cells expressing different cGAS variants. *H*, DR-GFP reporter assay was performed to examine the HR efficiency, as described in the “Experimental procedures” section. The represented data represented are shown as the means ± SDs of triplicate experiments. ∗∗*p* < 0.01. cGAS, cyclic GMP-AMP synthase; CPT, camptothecin; ETO, etoposide; HR, homologous recombination; HU, hydroxyurea; IR, ionizing radiation; MMC, mitomycin C; ns, nonsignificant.

The above data clearly demonstrate that SIRT3-mediated cGAS decrotonylation can hinder its binding to DNA and weaken its interaction with PARP1, which is important for HR repair. We investigated whether SIRT3 knockdown can sensitize cancer cells to IR. Colony formation assays demonstrated that HeLa cells expressing SIRT3-specific siRNA were significantly more sensitive to IR compared to controls, whereas SIRT3 overexpression did not significantly affect colony formation (Fig. 6, *A* and *B*). Knocking down cGAS or overexpressing PARP1 in SIRT3 knockdown cells resulted in a significant reduction in γ-H2AX levels starting at 2∼4 h postirradiation (Fig. S4*A*), suggesting that altering cGAS and PARP1 levels can partially mitigate DNA repair deficits caused by SIRT3 loss. Additionally, SIRT3 deficiency sensitized both HeLa and A549 cells to various DNA-damaging agents, including hydroxyurea (HU), camptothecin, etoposide (ETO), and mitomycin C (MMC) (Fig. 6, *C* and *D*). It also impaired the recruitment of key HR factors, RAD51 and RPA2, to DNA DSB sites (Figs. 6, *E* and *F* and S4, *B* and *C*). In cGAS K254R mutant cell lines, the addition of 3-TYP (SIRT3 inhibitor) did not affect the number of RAD51 and RPA2 foci (Figs. 4, *D* and *F* and S4, *E* and *G*). The addition of the 3-TYP further decreases the HR repair efficiency in the cGAS WT cell lines. However, the HR repair efficiency in the cGAS K254R mutant cell lines is not significantly affected by the addition of the SIRT3 inhibitor (Fig. 6, *G* and *H*). These results confirm that SIRT3 and cGAS decrotonylation are in the same pathway and indicate that SIRT3 is a potential target for cancer therapy.

## Discussion

In the complex narrative of cellular genome defense mechanisms (53), our study highlights a previously underexplored aspect: crotonylation of cGAS at K254 significantly influences DDR, especially in the context of IR-induced DNA damage. cGAS, which is well-known for its role in innate immunity, also plays a multifaceted role in DDR (54). Our findings reveal how its activity, mediated through PTMs such as crotonylation, extends beyond immune signaling to directly impact DNA repair processes.

cGAS responsiveness to DNA damage through changes in its crotonylation levels represents dynamic regulation, suggesting a finely tuned cellular mechanism that adjusts its activity in response to genomic stressors. The role of cGAS crotonylation in DNA repair not only highlights the complexity of cellular responses to DNA damage but also opens new avenues for exploring regulatory pathways in genome maintenance.

SIRT3, an NAD+-dependent protein deacetylase (or decrotonylase), regulates adaptive responses to stresses (55). Mitochondrial-localized SIRT3 regulates mitochondrial metabolism *via* its deacetylation activity (56). SIRT3 is also found in the nucleus, where it decrotonylates H3K4cr (38). While SIRT3 is implicated in various biological processes, its role in DSB repair and IR-induced DDR remains poorly understood (57, 58).

Elucidating the roles of CBP and SIRT3 in cGAS crotonylation has been a pivotal part of our research. CBP, the primary crotonylase, and SIRT3, the decrotonylase, are key influencers of its interactions with DNA and other integral proteins in the DNA repair process, such as PARP1. SIRT3 modulates cGAS–DNA complex formation in a dose-dependent manner, underscoring the significance of these regulatory interactions in the DDR.

The present study revealed the influence of SIRT3-mediated decrotonylation on DNA repair pathways. By inhibiting cGAS binding to DNA, SIRT3-mediated decrotonylation affects its interaction with PARP1 and indirectly promotes HR. This finding is particularly intriguing when juxtaposed with Liu *et al.*'s study, while our research indicated that decreased cGAS crotonylation facilitates HR repair, Liu *et al.* demonstrated a contrasting role of nuclear cGAS in suppressing DNA repair, highlighting its involvement in cancer progression (27). Our findings, along with those of Liu *et al.*, illustrate a complex, dual role of cGAS in DNA repair, indicating that the crotonylation status of cGAS may act as a molecular switch in HR repair processes. Our findings contribute to a deeper understanding of the decision-making processes involved in cellular DNA repair mechanisms.

The sensitivity of SIRT3-deficient cells to IR and other DNA-damaging agents represents a promising therapeutic strategy, particularly in the context of cancer treatment (59). Targeting the cGAS crotonylation–decrotonylation cycle could be a novel approach to sensitize cancer cells to radiation and chemotherapeutic agents, especially in cancers characterized by impaired DDR pathways.

Furthermore, the roles of cGAS in maintaining genome stability and the immune response highlight novel therapeutic avenues for diseases associated with immune dysregulation and genomic instability. Investigating the modulation of cGAS activity *via* PTMs, particularly crotonylation and decrotonylation, could lead to the development of innovative drugs.

In conclusion, the findings of our study hold potential for application in the development of new therapeutic strategies. By elucidating the role of cGAS crotonylation in regulating DNA repair pathways, especially under IR stress, and exploring the functional dynamics of cGAS, CBP, and SIRT3, this study paves the way for novel research avenues and therapeutic innovations. The insights provided by our study are crucial for understanding and addressing diseases related to genomic instability, particularly in the realm of cancer therapy.

## Experimental procedures

### Cell culture and transfection

All experimental cell lines, procured from the American Type Culture Collection (ATCC), include HEK293T (human embryonic kidney epithelial cell line), HeLa (human cervical cancer cell line), and A549 (human lung carcinoma cell line) cells, were cultured in Dulbecco’s Modified Eagle’s Medium supplemented with 10% (v/v) heat-inactivated fetal bovine serum and 1% (v/v) penicillin–streptomycin. All cultured cells were grown at 37 °C in a humidified incubator containing 5% CO2. All cell lines tested negative for *mycoplasma* contamination. Lipofectamine 2000 (Invitrogen, 11668027) was utilized for transfection in accordance with the manufacturer’s instructions.

### Antibodies and chemical reagents

The antibodies utilized in this study were as follows: anti-Crotonyllysine Mouse mAb (PTM-502, PTM BIO), anti-Acetyllysine Rabbit mAb (PTM-105RM, PTM BIO), anti-Flag (F3165, Sigma-Aldrich), anti-Flag@M2 Affinity Gel M (AZ220, Sigma), anti-GAPDH Rabbit mAb (PTM-5375, PTM BIO), anti-GFP (sc-9996, Santa Cruz Biotechnology), anti-alpha Tubulin (ab 7291, Abcam), anti-HA (sc-7392, Santa Cruz Biotechnology), anti- Rabbit IgG antibody (3900, Cell Signaling Technology), anti-Mouse IgG antibody (5415, Cell Signaling Technology), anti-CBP (sc-7300, Santa Cruz Biotechnology), anti-cGAS (83623, Cell Signaling Technology), anti-beta Actin antibody (ab8827, Abcam), anti-Myc (16286-1-AP, Proteintech), anti- SIRT3 antibody (5490, Cell Signaling Technology), anti-PARP1 antibody (39561, Active Motif), anti-γH2AX (05–636, Millipore), anti-RAD51 (ab 133534, Abcam), anti-RPA2 (ab 76420, Abcam), Alexa Fluor 488-labeled Goat Anti-Mouse IgG (H + L)(A-21202, Invitrogen), Alexa Fluor 568-labeled Goat Anti- Rabbit IgG(H + L)(A-11036, Invitrogen), Recombinant human CBP-HAT protein (ab167964, Abcam), Recombinant human SIRT3 protein (ab125810), Protein A/G PLUS-Agarose (sc-2003, Santa Cruz Biotechnology, Abcam), Streptavidin Agarose (20353, ThermoFisher Scientific). Annexin V, FITC Apoptosis Detection Kit was purchased from Dojindo. Campathecin (C9911), Hydroxyurea (400046-5GM), ETO (E1383), and MMC (M0503) were purchase from Sigma-Aldrich. NAM, TSA, 3-TYP, and protease inhibitor cocktail purchase from Selleck. DAPI (ZLI-9557) was purchased from ZSGB-BIO. DH5α(TSC-C14) and BL21(TSC-E01) were purchased from Tsingke Bio. Crotonoyl coenzyme A (28007-5MG) was purchased from Sigma. Cell Counting Kit (CCK-8) (40203ES) was purchase from Yeasen. PI/RNase Staining Solution (CY001-L) was purchase from SIMUBIOTECH. LightShift Chemiluminescent EMSA Kit (20148) was purchased from ThermoFisher Scientific.

### Plasmids and siRNAs

Flag-tag plasmids including CBP, p300, GCN5, PCAF, and S-protein-FLAG-Streptavidin-binding peptide–TIP60 were gifted from Jiadong Wang’s lab (Peking University Health Science Center). HA-CBP, GFP-cGAS, Flag-cGAS, NHEJ-GFP, DR-GFP, I-SceI, and pCherry plasmids are retained in our laboratory. Myc-tagged-SIRT1, SIRT2, and SIRT3 plasmids were generated by PCR amplification prior to their insertion into the pcDNA3.1-Myc vector. Glutathione S-transferase (GST)-cGAS and GST-cGAS K254R cDNAs were inserted into GST-pET-4T-1 to achieve GST-tag expression. GFP-cGAS and Flag-cGAS point mutations and Flag-cGAS truncated forms were synthesized by Taihe Biotechnology Co, Ltd. The biotinylated ISD was synthesized by Sangon Biotech. The accuracy of all constructs was confirmed by sequencing.

Synthesis of siRNAs was accomplished by the GenePharma Biotech (Shanghai). Regarding the transfection procedure, the indicated siRNA was used to accomplish twice transfection of cells with Lipofectamine 2000 (Invitrogen) at a 24-h interval as per the protocol of manufacturer. The siRNA sequences are listed as follows:

CBP-siRNA1: GCAAGAAUGCCAAGAAGAA;

CBP-siRNA2: GAUGCUGCUUCCAAACAUA;

SIRT3-siRNA1: GAAACUACAAGCCCAACGU;

SIRT3-siRNA2: ACGUUGGGCUUGUAGUUUC.

### Co-immunoprecipitation and Western blot

For the Co-IP assays, cells were lysed in NETN buffer (containing 300 mM NaCl, 20 mM Tris-base, 1 mM EDTA and 0.5% (v/v) NP-40) involving 1-fold protease inhibitor cocktail. After 20 min of continuous agitation at 4 °C, the cell lysates were centrifuged at 12,000 rpm for 10 min to collected the supernatant. This supernatant was then incubated with either beads or antibodies at 4 °C for 6 h. Following incubation, the samples were washed three times with NETN buffer containing protease inhibitors. The final immunoprecipitants were analyzed by immunoblotting. For the immunoblotting, protein samples (either lysate or precipitates) were denatured in 1x sodium dodecyl sulfate (SDS) protein sample buffer at 100 °C for 8 min, then resolved by electrophoresis through either a 6% or 8% SDS-polyacrylamide gel. The separated proteins were transferred onto polyvinylidene difluoride membranes and incubated with predefined antibodies at the indicated dilutions. Detection was performed using an enhanced chemiluminescence reagent (Thermo Fisher Scientific).

### Mass spectrometry

HeLa protein lysates were used in IP experiments with IgG or cGAS antibody. cGAS protein was eluted from beads and subjected to LC–MS/MS analysis. Prepare mobile phase A solution (100% water, 0.1% formic acid) and B solution (80% acetonitrile, 0.1% formic acid). A 1 μg sample of the supernatant was injected for liquid chromatography quality testing. Briefly, the mass spectrometer was operated in “top-40” data-dependent mode, collecting MS spectra in the Orbitrap mass analyzer (120,000 resolution, 350–1500 m/z range) with an automatic gain control target of 3E6 and a maximum ion injection time of 80 ms. The most intense ions from the full scan were isolated with an isolation width of 1.6 m/z. Following higher-energy collisional dissociation with a normalized collision energy of 27, MS/MS spectra were collected in the Orbitrap (15,000 resolution) with an automatic gain control target of 5E4 and a maximum ion injection time of 45 ms to generate raw mass spectrometry detection data (.raw).

### GST fusion protein purification

To purify GST fusion proteins, we followed the protocols provided by the manufacturer of the GST-tagged protein purification kit (Beyotime). GST proteins were produced from 1 L of BL21 competent cells cultured in LB medium. We induced protein expression in log-phase cultures (*A*_600_ ≈ 0.6) by shifting the temperature to 37 °C and adding 1 mM IPTG. After 3 h of vigorous shaking, cells were harvested by centrifugation at 5000 rpm at 4 °C for 10 min. The pelleted bacterial cells were lysed for 20 min and sonicated to disrupt cell membranes, and the insoluble material was removed by centrifugation. The soluble lysates were then incubated with BeyoGold GST-tag Purification Resin at 4 °C inside a tumbler. Following this, the resin was washed with a washing solution to remove any unbound material. Finally, the GST fusion proteins were eluted using an elution solution.

For the purification of cGAS and cGAS K254R proteins, solutions containing GST-cGAS and GST-cGAS K254R were prepared. PreScission Protease was added to these solutions at a ratio of 2 U per 100 μg of GST-tagged protein to cleave the GST tag from the fusion proteins. These mixtures were then incubated with pre-equilibrated BeyoGold GST-tag Purification Resin in PreScission Protease digestion buffer for 20 to 30 min at room temperature. After incubation, the mixtures were centrifuged at 500*g* for 5 min to separate the supernatant containing the target proteins from the undigested GST-tagged proteins and PreScission Protease, which remained bound in the gel.

### *In vitro* crotonylation and decrotonylation assay

An *in vitro* crotonylation assay was conducted using 1 μg of GST-cGAS WT or K254R (50 nM), 0.5 μg of CBP-HAT, and a mixture containing 300 μM crotonyl-CoA. This reaction mixture, totaling 50 μl, also included 50 mM Tris-Cl (pH 7.5), 100 mM NaCl, 1 mM EDTA, and 1 mM DTT. The reaction was carried out at 37 °C for 1 h. Subsequently, enzyme inactivation was performed by heating at 70 °C for 5 min. In addition, 0.2 μg of SIRT3 was mixed with the samples and incubated at 37 °C to achieve decrotonylation. SDS-PAGE was subsequently performed for sample separation, while an anti-Kcr antibody was used for Western Blotting for sample detection.

### Electrophoretic mobility shift assay

GST-cGAS WT and GST-cGAS K254R proteins were expressed and purified from BL21 *E. coli*. Subsequently, an EMSA was performed using the Electrophoretic Mobility Shift Assay Kit (Thermo Fisher #20148), following the manufacturer’s protocol. Briefly, the specified concentrations of proteins were mixed with biotin-labeled DNA (2 fM) substrate in a 20 μl reaction mixture containing 2.5% glycerol, 5 mM MgCl2, 50 ng/μl poly(dI.dC), and 0.05% NP-40. The samples were then incubated at room temperature for 20 min. Finally, the samples were separated on 6% native polyacrylamide gels in 0.5 × TBE buffer (2 mM EDTA, 90 mM Tris-HCl, 90 mM boric acid, pH 8.3), and the complexes were detected using an HRP-conjugated biotin antibody with the GE ImageQuant LAS 500 Imaging System.

### DNA pull-down assay

Cells were collected and lysed with NETN buffer (with protease inhibitor). Debris was removed by centrifugation, and 60 μl of supernatant was kept as whole-cell lysate. Five micrograms of biotin-ISD or label-free ISD (as a control) was added to the remaining supernatants and incubated at 4 °C for 4 h, followed by the addition of streptavidin beads for 2 h. Beads bound with proteins were washed three times with NETN buffer, resuspended in SDS–PAGE loading buffer, denatured, and analyzed by immunoblotting.

### Laser microirradiation assay

HeLa cells were plated in 35-mm glass bottom dishes (MatTek Corporation) and transfected with GFP-cGAS and GFP-cGAS K254R plasmids. Laser microirradiation was performed using a UV laser at 365 nm on a NIKON Ti2 microscope equipped with an Ilas Pulse (GATACA SYSTEMS). The laser output was set to 60% to ensure consistent generation of focused GFP stripes. Images were captured every 10 s for 10 min following the damage using Micro Manager software. Quantification of GFP localization (signal intensity of GFP at the microirradiation bands) was performed using Image J software. For each condition, 20 biological replicates were conducted.

### Generation of cGAS WT and cGAS K254R mutant cell lines

To generate the cGAS WT and cGAS K254R mutant cell lines, HeLa cells were initially transfected with cGAS shRNA (sequence: AGTTCTTACTGAAAAACAG) using a lentiviral system. Following transfection, cells were selected with 1 μg/ml puromycin. Two weeks after transfection, the knockdown efficiency was detected by Western blotting. Subsequently, these cGAS knockdown cell lines were transfected with Flag-pcDNA3.1-cGAS WT or K254R shRNA–resistant plasmids. Selection continued for 1 month using 0.5 mg/ml G418. Finally, successful expression of the constructs was confirmed by Western blotting using cGAS and Flag antibodies.

### NHEJ assay

Before transfection, a NHEJ-GFP plasmid was digested with HindIII enzyme overnight at 37 °C and recovered using AxyPrep DNA Gel Extraction kit (Axygen; Corning, Inc), according to the manufacturer's instructions. The cells were transfected with 0.25 μg of pCherry and 1 μg of the digested NHEJ-GFP plasmid and mixed with 2.5 μl of Lipofectamine 2000. Following 6 h, the culture medium of the transfected cells was replaced with fresh medium and further cultured for 2 days (60). The cellular fluorescence was measured by flow cytometry analysis, and the proportion of GFP-positive cells among the RFP-positive cells was calculated as the NHEJ repair efficiency, while the repair frequency is presented as the means ±SDs from three or more assays.

### HR assay

The cells were transfected with a single copy of a DR-GFP and I-SceI expression plasmid and with a pCherry plasmid used as a transfection efficiency control. Following 6 h, the culture medium of the transfected cells was replaced with fresh medium and further cultured for 2 days. The cellular fluorescence was measured by flow cytometry analysis, and the GFP-positive cell population was measured as the HR repair efficiency, while the repair frequency is presented as the means ±SDs from three or more assays.

### Flow cytometry

For the analysis of NHEJ and HR assays, cells were trypsinized with 0.25% trypsin and resuspended in PBS. Cellular fluorescence was then measured using a NovoCyte flow cytometer (ACEA Biosciences, Inc), with data analysis conducted *via* NovoExpress 1.3.0 software (ACEA Biosciences, Inc). For apoptosis analysis, following trypsinization, cells were washed with prechilled PBS and stained using the Annexin V-FITC Apoptosis Detection Kit according to the manufacturer's instructions. Fluorescence-activated cell sorting analysis was performed to determine the percentage of apoptotic cells, utilizing the same flow cytometry equipment.

### Immunofluorescence assay

Cells were seeded on coverslips in 6-well plates and transfected with the nontargeting siRNA or SIRT3 siRNA, and cells were accomplished using IR at a dose of 8 Gy. After removing the supernatants, cells were washed with PBS, fixed with 4% paraformaldehyde, permeabilized with 0.2% (v/v) TritonX-100, and blocked in 3% (w/v) BSA for 1 h. Then, the cells were incubated with the indicated primary antibodies (in 3% (w/v) BSA) overnight at 4 °C followed by incubation with fluorescent secondary antibodies and DAPI for 1 h at room temperature. Finally, placement of coverslips was accomplished on antifade buffer-containing slides, and a fluorescence microscope (Nikon) was utilized for result visualization.

### Clonogenic assay

Cells were serially diluted and plated. After 2 weeks of culture in an incubator, cells were stained with Coomassie reagent (ratio, methanol: acetic acid: Coomassie: H_2_O = 50:10:0.25:40) for 3 h. After staining, the cells were thoroughly washed with distilled water. Colonies consisting of at least 50 cells were counted to assess clonogenic survival. The survival rates were then calculated by comparing the number of colonies formed to the number of cells initially seeded.

### Cell viability assay

HeLa cells (2000 cells/well) were seeded into 96-well plates and subsequently treated with HU at 1 mM, ETO at 100 nM, camptothecin at 1 μM, or MMC at 5 μM. Twenty-four hours after treatment, cell viability was assessed by adding CCK8 reagent to each well. The absorbance was measured to analyze cell viability. Data are presented as the means ± SDs of at least triplicate experiments.

### Statistical analysis

Data for each group were analyzed using a two-tailed unpaired Student's *t* test with GraphPad Prism software (version 8.02) and are expressed as means ± SD, unless otherwise specified. Detailed statistical parameters, including significance levels, are provided in the figures and corresponding figure legends. Here, 'ns' denotes nonsignificant differences, while asterisks indicate levels of statistical significance: ∗*p* < 0.05, ∗∗*p* < 0.01, ∗∗∗*p* < 0.001.

## Data availability

All the data described in the article are within the article.

## Supporting information

This article contains supporting information.

## Conflict of interest

The authors declare that they have no conflicts of interest with the content of this article.
