# Supplementary material for: Decrotonylation of cGAS K254 prompts homologous recombination repair by blocking its DNA binding and releasing PARP1
Source: J Biol Chem. 2024 Jul 11;300(8):107554. doi: 10.1016/j.jbc.2024.107554 (PMC11345394; doi:10.1016/j.jbc.2024.107554)
Supplement: Supporting Information [file mmc1.pdf]

A

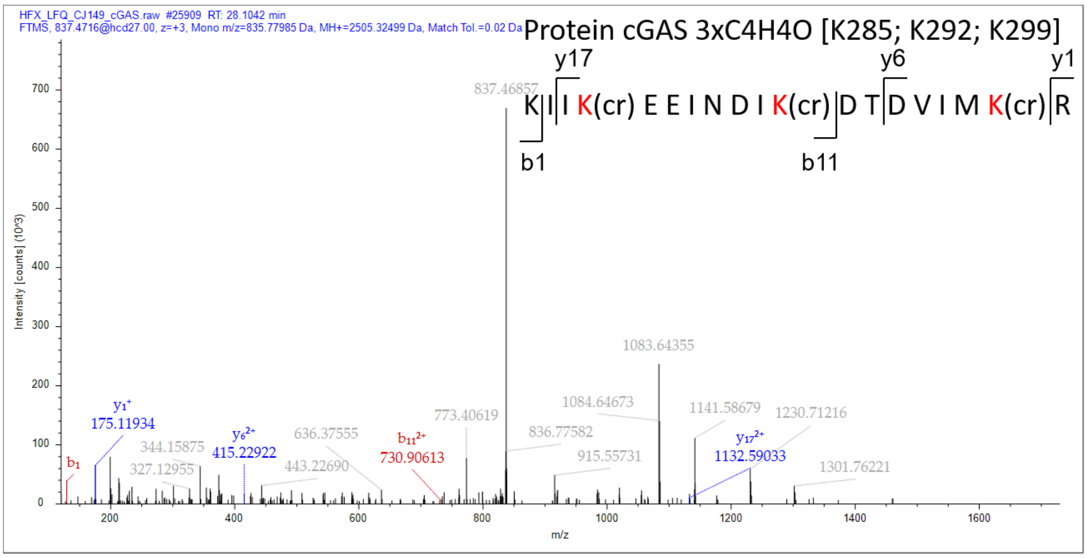

B

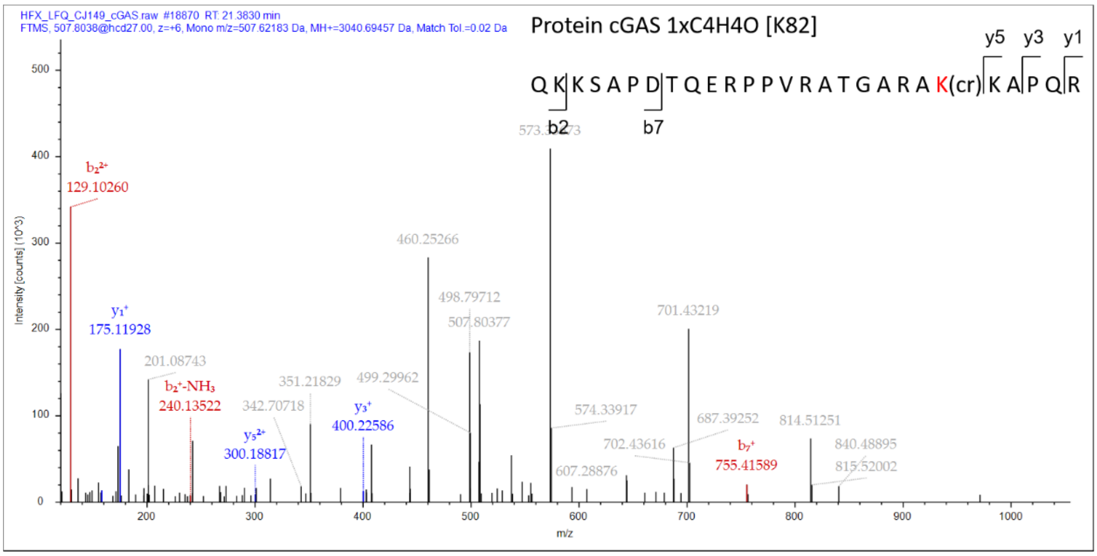

Fig. S1. *A* and *B*. Mass spectrometry analysis of cGAS crotonylation sites. The spectrum displays the mass-to-charge (m/z) ratios corresponding to peptide fragments containing crotonylated lysine, pinpointing the precise modification sites.

A

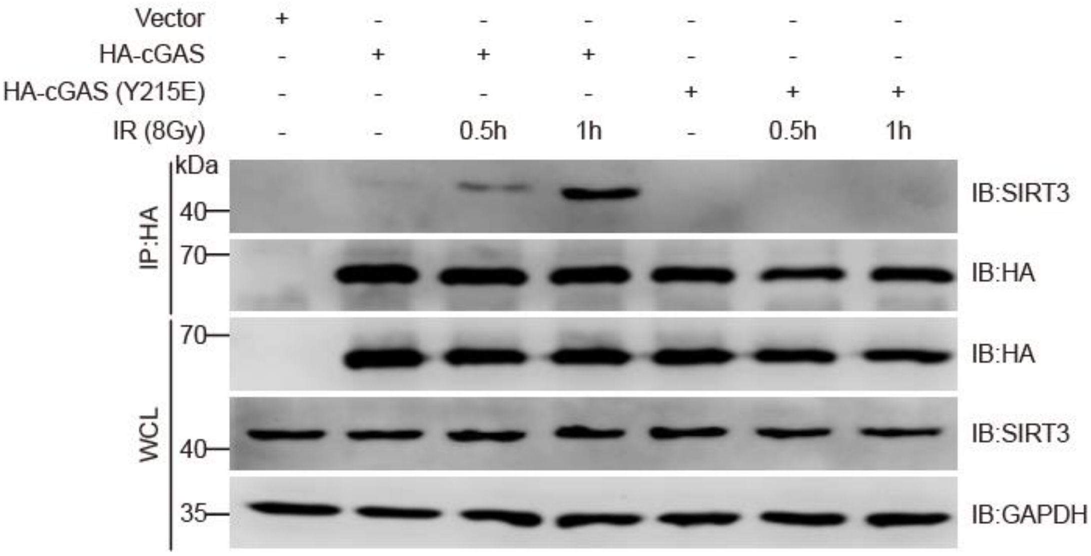

Fig. S2. *A*. HeLa cells were transfected with the indicated plasmids. Co-IP of endogenous SIRT3 with HA-cGAS and HA-cGAS Y215E from HeLa cells at the indicated times post-IR (8 Gy γ-ray).

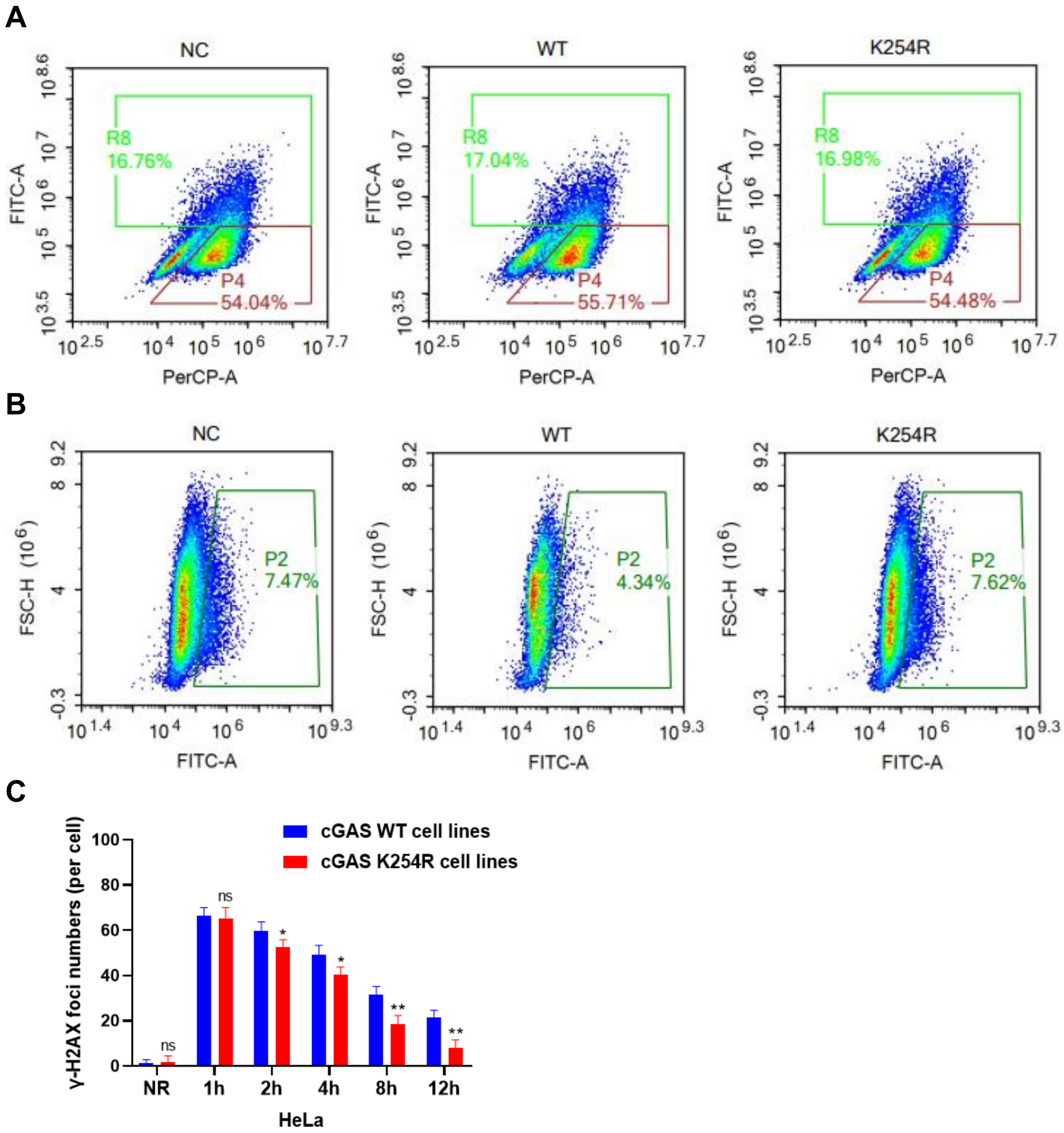

Fig. S3. *A* and *B*. Flow cytometry analysis of NHEJ repair efficiency and HR repair efficiency in cells expressing different cGAS variants. Data are representative of three independent experiments. *C*. Quantitative analysis of γ-H2AX foci in cGAS wild-type and cGAS K254R mutant cell lines at the indicated time points post-irradiation. Data represent the mean ± standard deviation (SD) of γ-H2AX foci per cell from three independent experiments, ns = nonsignificant, *$p < 0.05$, **$p < 0.01$.

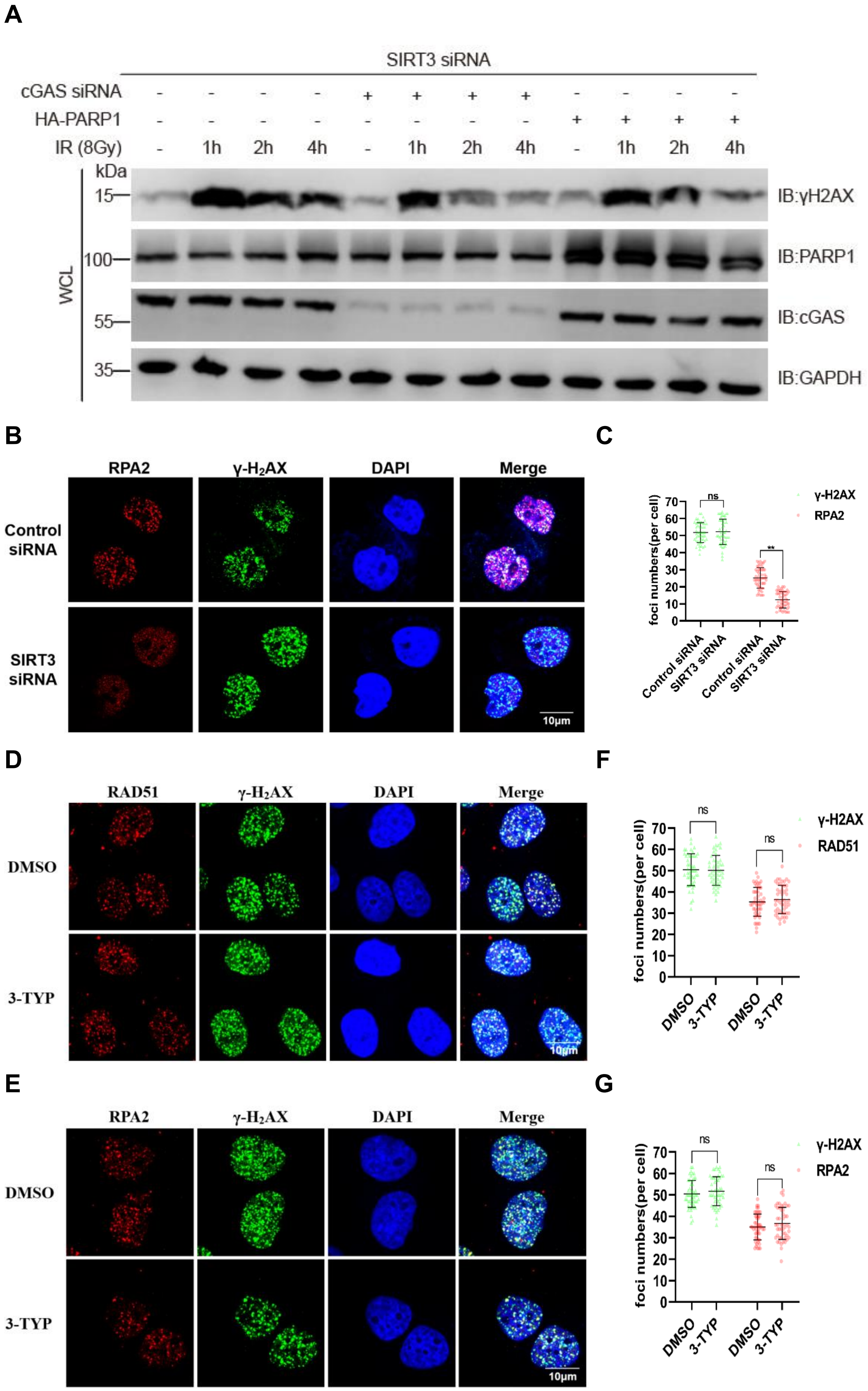
A
SIRT3 siRNA
cGAS siRNA
HA-PARP1
IR (8Gy)
1h
2h
4h
kDa
15
100
55
35
WCL
IB:γH2AX
IB:PARP1
IB:cGAS
IB:GAPDH
B
RPA2
γ-H2AX
DAPI
Merge
Control siRNA
SIRT3 siRNA
10μm
C
foci numbers(per cell)
γ-H2AX
RPA2
ns
**
Control siRNA
SIRT3 siRNA
D
RAD51
γ-H2AX
DAPI
Merge
DMSO
3-TYP
10μm
F
foci numbers(per cell)
γ-H2AX
RAD51
ns
DMSO
3-TYP
E
RPA2
γ-H2AX
DAPI
Merge
DMSO
3-TYP
10μm
G
foci numbers(per cell)
γ-H2AX
RPA2
ns
DMSO
3-TYP

Fig. S4. *A*. SIRT3 knockdown cells were further treated with siRNA to knockdown cGAS and transfected to overexpress PARP1. The efficiency of double-strand break (DSB) repair was assessed by monitoring γ-H2AX levels at various time points post-irradiation. *B* and *C*. HeLa cells were transfected with the indicated SIRT3 siRNAs. After 24 h, cells were treated with IR (8 Gy) for 1 h. An immunofluorescence assay was performed to test the entent of RPA2 foci formation using the corresponding antibodies. Scale bar, 10 μm. The data are indicated as means ±SD of triplicate, ns = nonsignificant, $^{**}p < 0.01$. *D* and *E*. HeLa cGAS-K254R mutant cell line was treated with the SIRT3 inhibitor 3-TYP. After 12 h, cells were treated with IR (8 Gy) for 1 h. An immunofluorescence assay was performed to test the entent of RAD51 and RPA2 foci formation using the corresponding antibodies. Scale bar, 10 μm. *F* and *G*. Quantitative analysis of RAD51 and RPA2 foci per cell in cGAS-K254R mutant cell line at indicated time points post-irradiation, with and without 3-TYP treatment. The data are indicated as means ±SD of triplicate, ns = nonsignificant.
